# Spatiotemporal changes of bacterial communities during a cyanobacterial bloom in a subtropical water source reservoir ecosystem in China

**DOI:** 10.1038/s41598-022-17788-7

**Published:** 2022-08-26

**Authors:** Zhenhua Huang, Cancan Jiang, Shengjun Xu, Xiaoxu Zheng, Ping Lv, Cong Wang, Dongsheng Wang, Xuliang Zhuang

**Affiliations:** 1 Key Laboratory of Environmental Biotechnology, Research Center for Eco-Environmental Sciences, Chinese Academy of Sciences, Beijing, 100085 China; 2 Yangtze River Delta Research Center for Eco-Environmental Sciences, Yiwu, 322000 China; 3 Institute of Tibetan Plateau Research, Chinese Academy of Sciences, Beijing, 100101 China; 4 University of Chinese Academy of Sciences, Beijing, 100049 China

**Keywords:** Microbiology, Environmental sciences

## Abstract

Cyanobacterial blooms, which not only threaten the health and stability of aquatic ecosystems but also influence the microbial community within, emerges as one of the most concerning problems in China. However, how cyanobacterial blooms affect the spatiotemporal variation of aquatic microbial communities remains relatively unclear. In this study, we used high-throughput sequencing to investigate how the cyanobacterial and bacterial community spatiotemporally vary along with main cyanobacterial bloom phases in upstream rivers of a eutrophicated water source reservoir. Both cyanobacterial and bacterial diversities in each river were significantly lower (*P* < 0.05) during the bloom outbreak phase, showing the apparent influence of cyanobacterial bloom. Dominant cyanobacterial taxa included *Cyanobacteriales* and *Synechococcales*, and dominant bacterial taxa comprised *Acinetobacter*, CL500-29, *hgc*I clade, *Limnohabitans*, *Flavobacterium*, *Rhodoluna*, *Porphyrobacter*, *Rhodobacter*, *Pseudomonas*, and *Rhizobiales*, whose changes of relative abundance along with the bloom indicated distinct community composition. Non-metric multidimensional scaling analysis proved that community composition had significant difference amongst bloom phases. Linear discriminant analysis (LDA) with LDA effect size analysis (LEfSe) identified unique dominant cyanobacterial and bacterial OTUs at different phases in each river, indicating spatiotemporal variations of communities. Canonical correlation analysis or redundancy analysis revealed that at different bloom phases communities of each river had distinct correlation patterns with the environmental parameters (temperature, ammonium, nitrate, and total phosphorus etc.), implying the spatial variations of microbial communities. Overall, these results expand current understanding on the spatiotemporal variations of microbial communities due to cyanobacterial blooms. Microbial interactions during the bloom may shed light on controlling cyanobacterial blooms in the similar aquatic ecosystems.

## Introduction

The frequency and intensity of cyanobacterial blooms are increasing in many inland aquatic ecosystems, including rivers, lakes, and reservoirs, due to excessive nitrogen and phosphorus enrichment in surface waters over-enrichment^1–4^. These blooms are becoming one of the top concerns of environmental protection agencies, water regulation authorities, and society, and the biodiversity of aquatic ecosystems is decreasing^5–7^. Furthermore, many bloom species can produce a variety of metabolites, which will bring dramatic health risks to humans and animals^8^. In China, to increase diversities of aquatic ecosystems, a series of water protection campaigns have been initiated in recent decades, which are further strengthened in the National Fourteenth Five Year Plan. The mechanisms of blooms outbreak, as well as corresponding counter-measures, have become one of the central research hotspots.

It has been universally acknowledged that the bloom species, such as cyanobacteria and other eukaryotic algae, are also an integral part of the microbial community within the aquatic ecosystems^9^, which not only rapidly respond to the changes of surrounding environmental parameters and form blooms but also actively interact with their bacterial counterparts. Previous studies on bacteria-cyanobacteria interaction focused on the composition and diversity of heterotrophic bacteria living with cyanobacteria (forming micro-niche or biofilms) as well as the differences between cyanobacteria-associated bacterial communities and the free-swimming communities^9–12^. The complex interactions between cyanobacteria-bacteria are crucial in ecological and biogeochemical terms, due to their fundamental functions in shaping aquatic communities^11^. It should be noted that the cyanobacterial blooms exhibit an ebb-and-flow pattern when dynamically responding to the changes of environmental parameters, therefore, the associated and the surrounding bacterial communities are presumed to be changing accordingly. However, the dynamic variations of bacterial communities in water bodies that served as drinking sources along the bloom course remains unclear.

In a previous study, we evaluated the spatiotemporal changes and eutrophic characteristics of water quality of a life-dependent reservoir (Yankou Reservoir Basin) with its upstream rivers, which served as drinking water sources for Yiwu City, Zhejiang Province of China^1^. Monthly water qualities assessment from 2013 to 2018 demonstrated that over 90% of the months the upstream rivers were collectively under eutrophic conditions, which kept aggravating eutrophic conditions of the reservoir. Before and during then, massive cyanobacterial blooms were observed in those upstream rivers and a large part of the reservoir infield, making it an immediate issue to be dealt with.

On the presumption that the bacterial communities would dynamically change along the cyanobacterial bloom course, the investigation into these variations will reveal which bacterial communities and how they will change, and the community information could eventually be used to develop potential predictive or mitigating tools against the bloom. For this purpose, surface water samples were collected from the four upstream rivers at different phases of full cyanobacterial blooms, and the composition and diversity of cyanobacterial and other bacterial communities of each river at each phase were investigated. The dynamic changes of cyanobacterial and bacterial communities along the bloom were revealed and correlated with the main environmental parameters.

## Materials and methods

### Sampling area and environmental parameters determination

**Figure 1:**
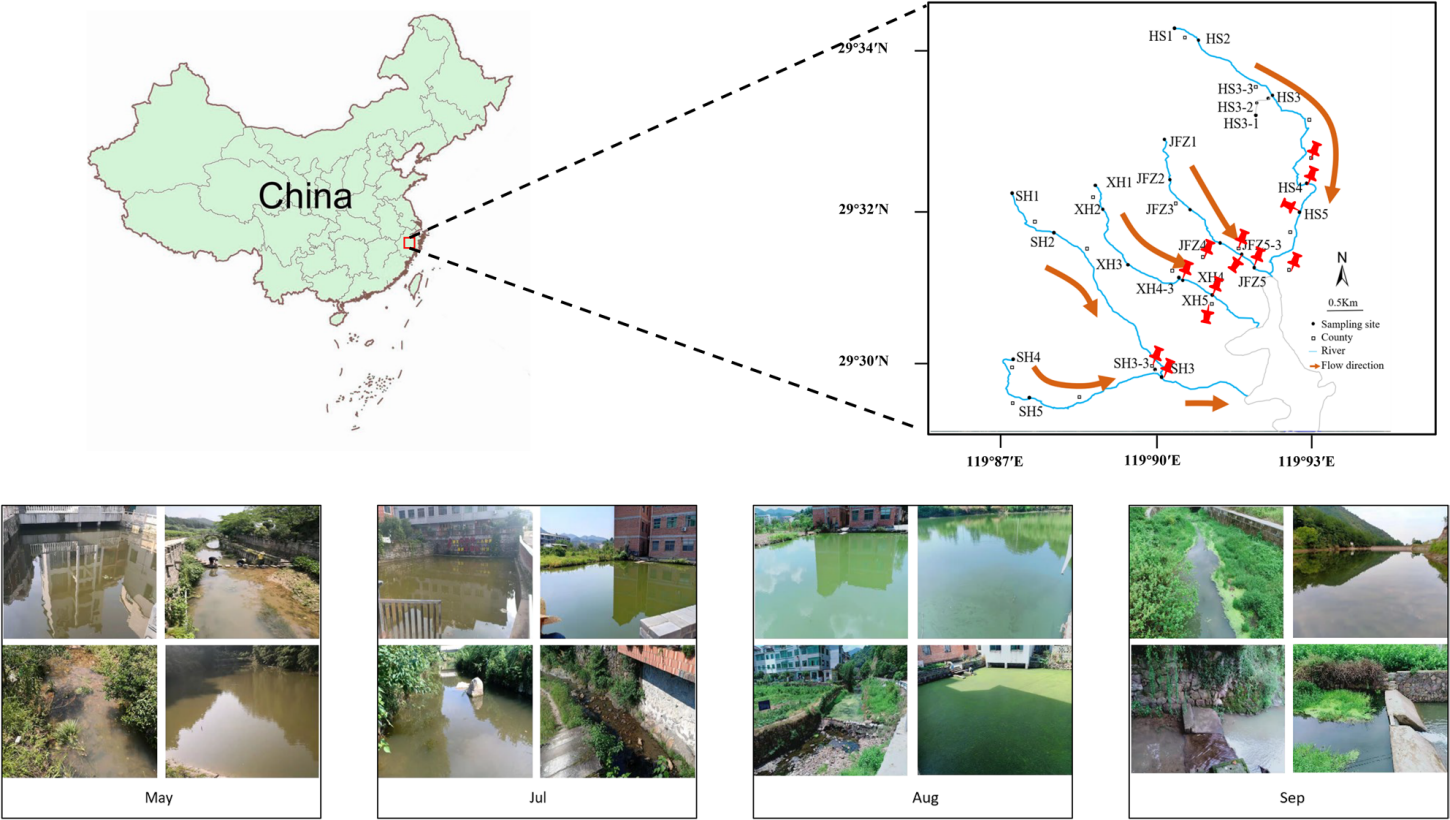
Diagram of Yankou Reservoir Basin indicating the sampling points on each river. The sampling sites were located using the global positioning system (GPS) and were nominated after the names of corresponding rivers. Map was created with ArcGIS software (V10.6, https://desktop.arcgis.com/zh-cn/desktop/). Field photos were taken at each sampling point (pins only indicated some of the locations for concise presentation), and representative ones were selected to show the bloom course from May to September.

This study was carried out along the four upstream rivers, namely Huangshan River (HS), Jinfuzhai River (JFZ), Sihe River (SH), and Xihua River (XH), of Yankou Reservoir basin (29° 17′ 25″ – 29° 18′ 49″ N, 119° 54′ 11″ – 119° 55′ 13″ E) in Yiwu City, Zhejiang province, middle east of China (Fig. 1). Based upon field observation of dynamics of a cyanobacterial bloom and Chil-*a* concentration determination, the outbreak course was primarily divided into four phases, including before the cyanobacterial bloom (Before AB), followed by early phase (Pre AB), outbreak phase (During AB), and late phase (Post AB). Additionally, each river has at least one tributary pool (HS has three pools) connecting to mainstream of the river; therefore, water samples would also be collected from the pools. A total number of 104 surface water samples (0.5 m depth) were collected, respectively in May, July, August, and September 2020 (26 samples for each sampling campaign), representing the four cyanobacterial bloom phases. The surface water samples were collected within a 3-day period in all the phases. After immediate collection, the samples were kept in an ice box filled with dry ice and transported back to the laboratory for subsequent treatment. Water samples (~ 3000 mL) were first passed through a 200-μm pore-size sieve to remove any debris for subsequent determination of water quality parameters and microbial community analysis. For bacterial and cyanobacterial community analyses, water samples (~ 1500 mL) were further filtered through 0.22-μm pore-size polycarbonate membranes (50 mm, Jinteng®, Tianjin, China), and those membranes were stored at − 80 °C until DNA extraction. The environmental parameters of the water samples were determined according to study described previously^13^. Briefly, a Hydrolab HQ30D multiparameter water quality meter (HACH Company Co., Loveland, the USA) was used in situ to monitor the water temperature (TEMP), oxidation–reduction potential (ORP), pH value, and chlorophyll-*a* (Chl-*a*). Other water quality parameters, including chemical oxygen demand (COD), total nitrogen (TN), dissolved total nitrogen (DTN), ammonium (NH_4_^+^), nitrite (NO_2_^−^), nitrate (NO_3_^−^), total phosphorus (TP), dissolved total phosphorus (DTP) and orthophosphate (MPO_4_^−^) were determined using the standard methods published by China’s State Environmental Protection Administration^14^.

### DNA extraction

The total DNA of microbial communities was extracted directly from the membrane filters using a FastDNA™ Spin Kit (MP Biomedicals, Santa Ana, CA, the USA) according to the manufacturer’s instructions. The purified total DNA was processed and sequenced targeting the V3–V4 region of 16S rRNA gene with 338F and 806R primers^15^ on MiSeq 300 platform by Shanghai Majorbio Bio-Pharm Technology Co., Ltd. The downstream alignment and analyses were performed using the integrated and free online platform of Majorbio Cloud Platform (www.majorbio.com).

### Bioinformatic treatment

The sequenced paired-end reads were merged with FLASH (v.1.2.11), and the raw reads were processed and analyzed using QIIEM (v.1.9.1) to remove low quality reads. After quality control, sequences were clustered into operation taxonomic units (OTUs) using UPARSE (v.7.0.1090) with a 97% sequence similarity threshold^16^. A representative sequence from each OTU was run against the databases of SILVA (Release 138). To avoid mistaken taxonomic alignments, the taxonomic classifications were double-checked against the reference prokaryotes. Those unclassified OTUs were discarded, followed by normalization of the filtered sequence data to minimize sequencing biases and allow for appropriate comparison of community variations.

For comparisons of microbial taxa variations across the cyanobacterial bloom phases, we defined those taxa with relative abundance less than 0.01% as rare taxa, more than 1% as abundant taxa, and between 0.01% and 1% as moderate taxa, respectively. Based upon the definition, those relevant taxa in subsequent analyses were categorized into groups as follows: always abundant taxa (AAT), always rare taxa (ART), always moderate taxa (AMT), and conditionally varied taxa (CVT), and the taxa with more than 0.01% relative abundance at certain phases in each sample were also referred as “dominant taxa” according to previous studies^17^.

### Statistical analyses

In this study, the bloom was mainly attributed to cyanobacterial overproduction, therefore, the cyanobacterial community would be further extracted from the normalized data for each sample, and alpha- and beta diversities were estimated both on cyanobacterial and the rest of bacterial communities (will be referred as “bacterial communities” below). Additionally, a comprehensive analysis on the changes of bacterial communities at different phases in each river as well as the variance of communities at the same phase amongst different rivers was performed.

To better compare the difference of communities on relative abundances and community compositions, both alpha- and beta-diversity indices were calculated. The alpha-diversity indices including OTU numbers (Sobs), abundance-based coverage estimator (ACE), Shannon, and Chao 1, as well as Venn diagrams compared to different samples were calculated using the *vegan* package in R language (v.4.0.0). Rarefaction curves and Good’s coverage were performed with MOTHUR (v.1.30.2). Significance amongst alpha-diversity indices were calculated using one-way ANOVA and Student’s *t*-test with the significant level at 0.05. For beta-diversity analysis, the non-metric multidimensional scaling analysis (NMDS) with weighted unifrac similarity coefficient and permutational multivariate analysis of variance test (PERMANOVA) was used to evaluate both the discrepancy of bacterial communities at different phases and the variance of communities at the same phase in different rivers.

To identify the cyanobacterial and bacterial OTUs contributing to the difference amongst samples described above, a significance test was first performed using the Kruskal–Wallis test coupled with Tukey–Kramer post-hoc examination, based upon OTUs’ relative abundances. Subsequently, the linear discriminant analysis (LDA) coupled with the LDA effect size (LEfSe) technique, with a discriminant analysis score of 2.0, was performed to identify the significantly different dominant OTUs amongst the samples. Additionally, cyanobacterial and bacterial OTUs with LDA scores over 3.0 and 4.0, respectively, were screened out to double-check with the significance tests. The significance tests and LEfSe analyses were all examined at a significant level of 0.05.

Finally, a variance inflation factor analysis (VIF) was first performed on all of the environmental parameters, only when VIF scores of the parameters were under 10, would the parameters be selected for subsequent correlation analyses. In this study, the VIF scores of DTN, DTP, and COD were over 10, which would be eliminated. Canonical correlation analysis (CCA) or redundancy analysis (RDA) was conditionally chosen based upon results of detrended correspondence analysis (DCA) to reveal the correlations.

## Results

### Environmental parameters of each river at different phases

Water samples were collected from each river, and complete environmental parameters were summarized in Table S1. In general, the Chl-*a* in the pools was higher than that in the corresponding rivers, and the maximum concentration reached 535.68 µg/L in JFZ’s pool. Further statistical analyses on the complete parameters of each river at different bloom phases indicated that for each river, there was no significant difference amongst the parameters at different phases (indicating as N.S.), except ORP and Chl-*a* (*P* < 0.05, Table S1). For these two parameters, detailed significances between relevant phases were included.

## General statistics of sequencing data and alpha-diversity comparison

### General statistics of sequencing data

In this study, a total number of 5,115,068 sequences was pooled for the total 104 samples, and sequencing data was normalized to 18,633 sequences for each sample, which were categorized into 8689 OTUs and 2998 bacterial species. The cyanobacterial community was collectively extracted from each sample, stratified into 515 OTUs and 191 species, leaving the bacterial communities constituting 8174 OTUs and 2807 species.

Rarefaction curves for each sample showed that most samples tended to approach saturation (Fig. S1), and the Good’s coverage ranged from 94.91 to 99.42% amongst the samples. The two indices indicated that the majority of the bacterial taxa had been extracted from the studied communities. Considering that the tributary pools were one of the main sources of cyanobacterial communities in rivers, alpha-diversity indices, including Shannon and Chao 1, were analyzed on cyanobacterial and the rest of bacterial communities in pools and rivers.

### Alpha-diversity comparisons and statistics

For cyanobacterial communities in pools of HS, JFZ, SH, and XH across the bloom phases, the Shannon index maximized at the “During AB” phase for JFZ and XH. Although it maximized at the “Post AB” and “Before AB” phase for HS and SH, respectively, the diversities at the “During AB” phase were also relatively high for both pools (Fig. S2A). Whilst Chao 1 index of these cyanobacterial communities showed a concurrent pattern that community diversities minimized at “During AB” and maximized at “Post AB” phase. For bacterial communities, both Shannon and Chao 1 indicated that community diversity decreased at “During AB” and increased afterward. On the contrary to pools, cyanobacterial communities in all rivers minimized at “During AB” phases according to Shannon and Chao 1 index (Fig. S2B), except for Shannon of HS and JFZ. Similar to pools, bacterial communities in all rivers were minimized at “During AB” phases and increased afterward based upon both indices estimates.

**Figure 2:**
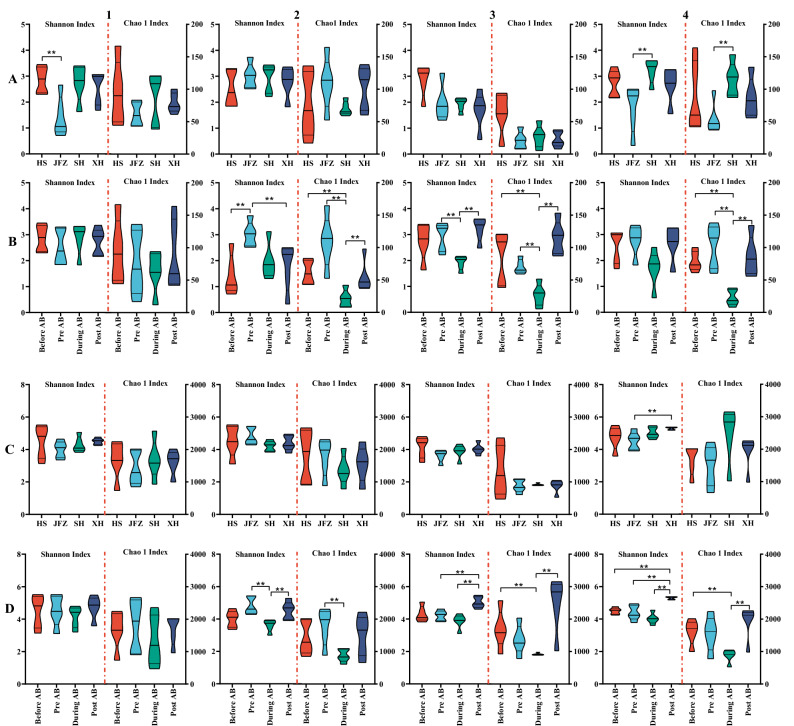
Comparisons of alpha-diversity estimators of Shannon and Chao1 on cyanobacterial (**A1**–**A4** and **B1**–**B4**) and bacterial communities (**C1**–**C4** and **D1**–**D4**). Figures of (**A1**–**A4**) illustrated cyanobacterial community diversity amongst different rivers at the same phases of Before AB (**A1**), Pre AB (**A2**), During AB (**A3**), and Post AB (**A4**), respectively; and (**B1-B4**) showed cyanobacterial community diversity of HS (**B1**), JFZ (**B2**), SH (**B3**), and XH (**B4**) at different bloom phases. (**C1**–**C4**) and (**D1**–**D4**) represented comparisons of bacterial communities equivalent to (**A1**–**A4)** and (**B1**–**B4**).

Differences on alpha-diversity were further determined amongst different rivers at the same phase as well as each river along the bloom phases (Fig. 2). For the cyanobacterial communities at each phase, there was, in general, no significant difference amongst the studied rivers (*P* > 0.05), except for the comparisons between HS and JFZ at “Before AB”, as well as JFZ and SH at “Post AB” phases (*P* < 0.05) (Fig. 2A). Whereas, community diversities in individual rivers exhibited apparent variances across the consecutive phases (Fig. 2B). Cyanobacterial communities in HS showed no significant difference across the phases, whilst communities in JFZ, SH, and XH demonstrated significant differences amongst these phases, and especially, communities at the “During AB” phase were significantly lower (*P* < 0.05) than the rest phases according to Chao 1 index for JFZ, SH, and XH.

Similarly, for bacterial communities at the same phase, there was, in general, no significant difference amongst the rivers (*P* > 0.05), except for the comparison between JFZ and XH according to Shannon index (Fig. 2C). Bacterial communities in individual rivers also showed similar variance with the cyanobacteria across the phases (Fig. 2D). HS communities had no significant difference amongst the phases (*P* > 0.05), whilst JFZ, SH, and XH communities showed significant differences, and communities at the “During AB” phase were also significantly lower (*P* < 0.05) than some of the rest phases in JFZ, SH, and XH, respectively.

OTU comparisons of both cyanobacteria and bacteria in tributary pools and their rivers at corresponding phases were performed (Fig. S3). In general, for both cyanobacterial and bacterial OTUs, the comparisons either amongst pools and rivers at certain phases (Fig. S3) or in each pool and river at different phases (Fig. S3), only a small portion of OTUs was shared in common amongst the four compared counterparts. It should be noted that only a small number of cyanobacterial OTUs was classified in pools of JFZ, SH, and XH during the bloom (Fig. S3), which supported the alpha-diversity analyses that the cyanobacteria diversities during the bloom were significantly lower than the rest phases for JFZ, SH, and XH (Fig. 2).

### Comparisons of cyanobacterial and bacterial community composition

**Figure 3:**
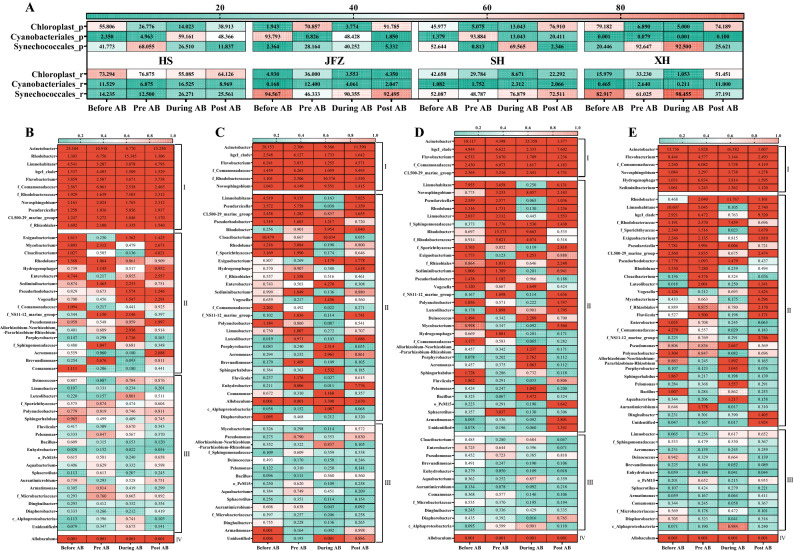
Dynamics of dominant cyanobacterial and bacterial taxa along with the cyanobacterial bloom. (**A**) Dominant cyanobacteria at order level in each tributary pool (_p) and rivers (_r). (**B**–**E**) Top 50 dominant bacterial genus in HS, JFZ, SH, and XH, respectively. Digits in boxes represented the relative abundances of cyanobacterial and bacterial taxa, and the group I-IV stood for always abundant taxa (AAT), conditionally varied taxa (CVT), always moderate taxa (AMT), and always rare taxa (ART), respectively. Bacterial taxa that categorized as “abundant” (> 1%) and “rare” (< 0.01) were shown in unified color.

In general, the most diverse cyanobacterial OTUs were assigned to phylogenetic orders of *Cyanobacteriales* and *Synechococcales* in both rivers and their tributary pools. However, the cyanobacterial community composition exhibited spatial variance amongst these ecosystems. In each pool, the 2 taxa took turns as the most dominant community, for example, in the tributary pool of HS, the most dominant community was *Synechococcales* and *Cyanobacteriales* across each of the phases; whilst JFZ had *Cyanobacteriales* as the most dominant community. And *Synechococcales* was the most dominant in the rest of the three rivers along with the bloom (Fig. 3A).

For bacterial communities, the abundant genus to which most diverse OTUs were assigned were sorted out in each river. Based upon the relative abundance of each taxon, the phylogenetic genera were further categorized into groups of AAT, ART, AMT, and CVT, respectively (Fig. 3B–E). Although these rivers had some genera in common in each group (AAT, ART, AMT, and CVT), such as *Acinetobacter* and *Flavobacterium* the bacterial community composition also exhibited spatial variance amongst the studied rivers. For example, a community of *hgc*I clade was always abundant in HS, JFZ, and SH, whilst was conditionally varied in XH, and its relative abundance was greatly reduced during the cyanobacterial bloom, especially in XH. Communities of *Pseudarcicella* and *Limnohabitans* in HS were always abundant, and their relative abundance increased during the cyanobaterial bloom, however, they were conditionally varied and noticeably reduced in the rest three rivers during the same phase. Similarly, *Comamonadaceae* was always abundant in each river, but its relative abundance clearly reduced during the bloom, and *Allobaculum* was always rare in HS, SH, and XH, but conditionally varied in JFZ that its relative abundance greatly increased during the bloom.

**Figure 4:**
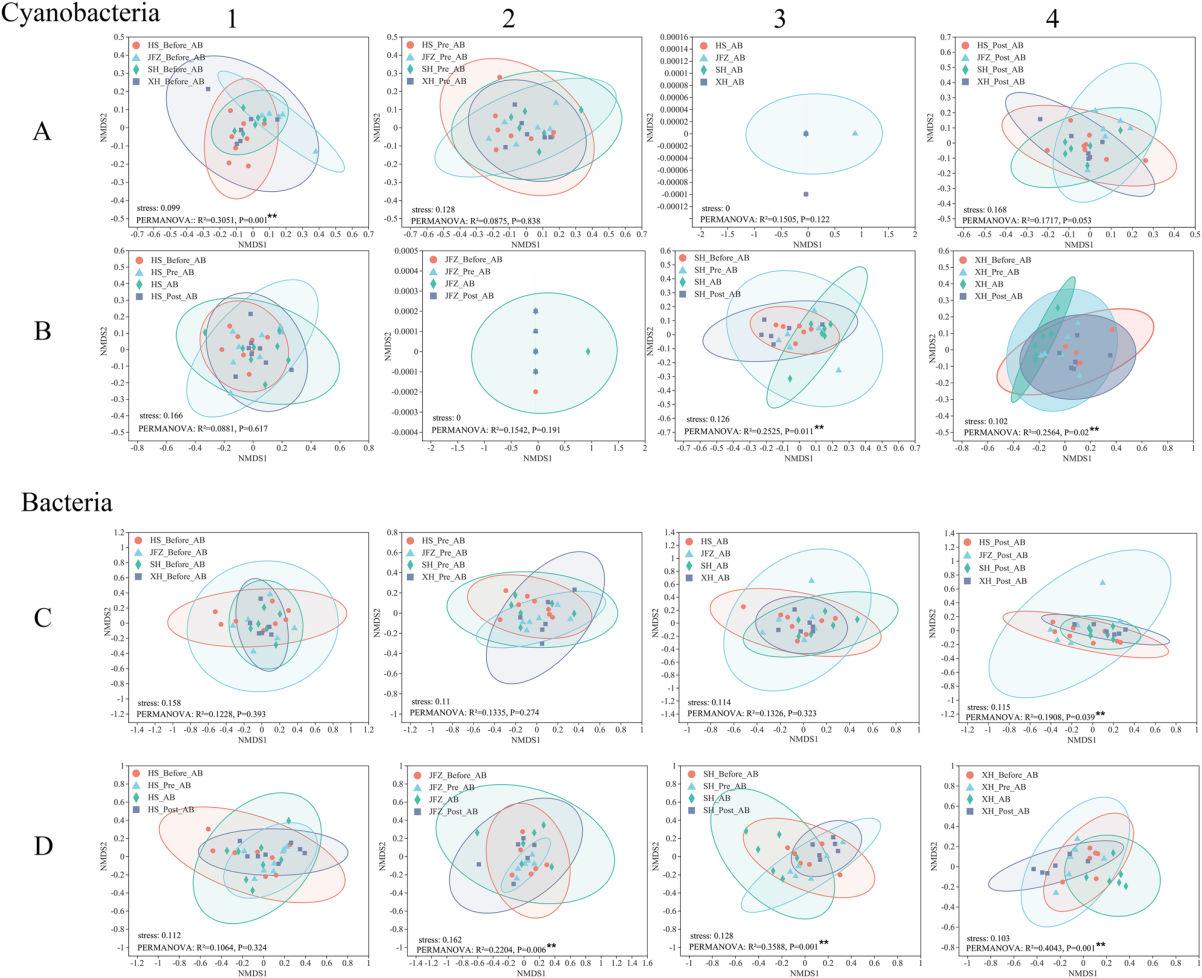
Non-metric multidimensional scaling analysis (NMDS) on cyanobacterial and bacterial communities. Figures of (**A1**–**A4**) presented the difference of cyanobacterial community in different rivers at the same phase, and figures of (**B1**–**B4**) illustrated the difference of cyanobacteria of each river at different phases. Figures of (**C1–C4**) and (**D1–D4**) were the bacterial communities equivalent to (**A1**–**A4**) and (**B1**–**B4**) comparisons, respectively. ** indicated the significant level at 0.05.

Variations of cyanobacterial and bacterial communities were further illustrated through NMDS with PERMANOVA test. Cyanobacterial communities at the “Before AB” phase exhibited significant variation amongst the studied rivers (PERMANOVA, df = 3, *F* model = 3.220, R^2^ = 0.305, *P* = 0.001), however not at the rest phases (Fig. 4A). Across the bloom phases, cyanobacterial communities in SH and XH showed significant variations (PERMANOVA, df = 3, *F* model = 2.252 and 2.298, R^2^ = 0.252 and 0.256, *P* = 0.011 and 0.02, respectively), whilst those in HS and JFZ did not (Fig. 4B). For bacterial communities, they only showed significant difference at the “Post AB” phase amongst the studied rivers (*df* = 3, *F* model = 1.729, R^2^ = 0.191, *P* = 0.039) (Fig. 4C). However, except for HS, bacterial communities in JFZ, SH, and XH collectively revealed significant discrepancy across the cyanobacterial bloom phases (*df* = 3, *F* model = 1.884, 3.731, and 4.525, R^2^ = 0.220, 0.359, and 0.404, *P* = 0.006, 0.001, and 0.001 respectively) (Fig. 4D1–D4).

### The significant difference in cyanobacterial and bacterial dominant species (OTUs)

The above results indicated that the discrepancies of both cyanobacterial and bacterial communities could be more attributed to the difference of cyanobacterial bloom phases instead of spatial heterogeneity of rivers. Therefore, the significantly different OTUs of cyanobacterial and bacterial communities were analyzed amongst samples at different phases in each river (containing corresponding tributary pools) (Table S2). The significance test was subsequently used for LEfSe analysis (Table S3) to identify those cyanobacterial and bacterial OTUs at different phases contributing to the significance.

**Figure 5:**
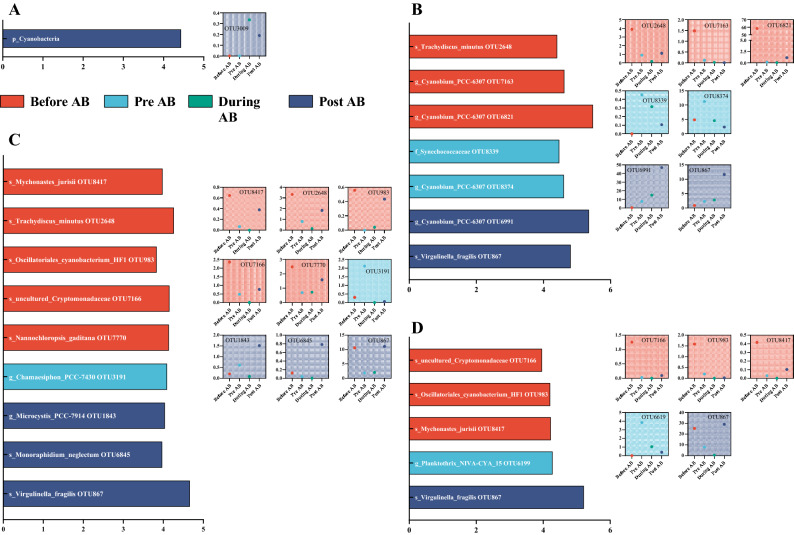
Significant cyanobacterial species (OTUs) based on significant and LEfSe analyses. Figures of (**A**–**D**) represented HS, JFZ, SH, and XH rivers. Bar plots exhibited LDA scores of each OTU, with its clearest phylogenetic level, and the scattered dot plots illustrated the dynamic changes of each OTU’s relative abundance.

For cyanobacteria, more OTUs were identified in JFZ, SH, and XH, and these OTUs have mainly identified at the rest three phases except the “During AB” phase (Fig. 5). Cyanobacterial OTUs at different phases were mainly classified into *Cyanobacteriales* (OTU1843), and *Leptolyngbyales* (OTU3191) in SH (Fig. 5C), and were pooled into *Cyanobacteriales* (OTU6199) in XH (Fig. 5D), respectively. Additionally, several OTUs, including OTU2648, OTU7367, OTU8417, and OTU867, were widely distributed amongst these three rivers, indicating that a core cyanobacterial community was shared in common in these rivers.

**Figure 6:**
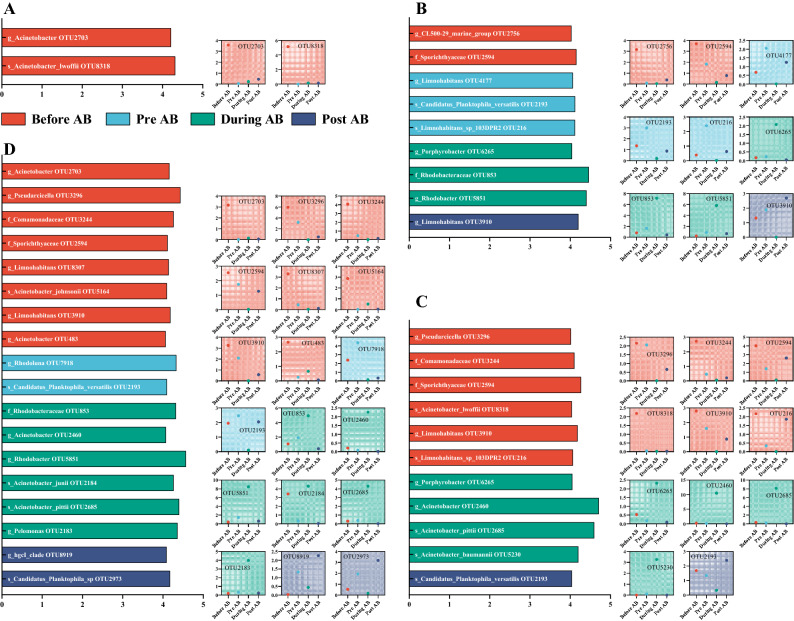
Significant bacterial species (OTUs) based on significant and LEfSe analyses. Figures of (**A**–**D**) represented HS, JFZ, SH, and XH rivers. Bar plots exhibited LDA scores of each OTU, with its clearest phylogenetic level, and the scattered dot plots illustrated the dynamic changes of each OTU’s relative abundance.

Similarly for the bacterial community, more OTUs were identified in JFZ, SH, and XH, however, were universally identified across the cyanobacterial bloom phases (Fig. 6). Members of *Rhodobacteraceae* (OTU 853 and OTU 5851), *Limnohabitans* (OTU4177, OTU216, and OTU3910), *Porphyrobacter* (OTU6265), and *Sporichthyaceae* (OTU2594) were mainly screened out in JFZ (Fig. 6B), whilst *Pseudarcicella* (OTU3296), *Comamonadaceae* (OTU3244), *Sporichthyaceae* (OTU2594), *Limnohabitans* (OTU3910), *Acinetobacter* (OTU8318, OTU2460, OTU2685, OTU5230, OTU2703, OTU5164, OTU483, and OTU2184) were broadly identified in SH and XH (Fig. 6C,D). Notably, members of *Rhodobacteraceae* (OTU853 and OTU5851), *Porphyrobacter* (OTU6265), and *Acinetobacter* (OTU2460, OTU2685, OTU2184, and OTU5230) in JFZ, SH, and XH exhibited significantly higher (*P* < 0.05) relative abundances at “During AB” phase than the rest three phases (Fig. 6B–D), which may indicate their relationships with the bloom-forming cyanobacterial communities.

**Figure 7:**
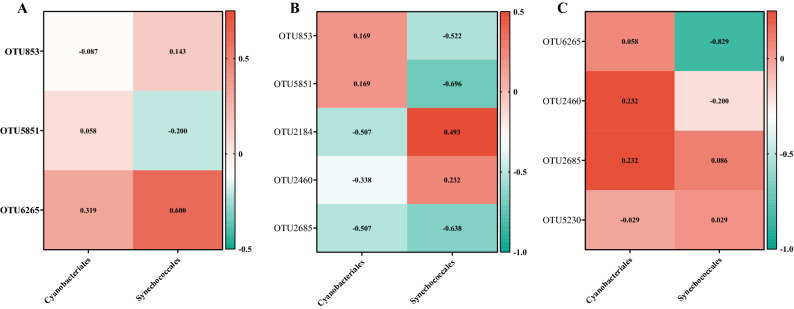
Correlation analysis between dominant cyanobacterial taxa and significant bacterial species (OTUs) identified in JFZ (**A**), SH (**B**), and XH (**C**). Digits in boxes of the heatmap represented the correlation coefficients.

Correlations between the dominant cyanobacterial taxa and the above-identified dominant species were subsequently analyzed (Fig. 7). In general, the results indicated that some bacterial taxa in different aquatic ecosystems showed different correlations with the same cyanobacterial taxa. For example, OTU853 (*Rhodobacter*) in JFZ was negatively correlated with *Cyanobacteriales* along with the whole bloom phases (Fig. 7A), however, it showed contrasting correlation patterns with *Cyanobacteriales* in XH (Fig. 7C). OTU6265 (*Porphyrobacter*) in JFZ was positively correlated with *Cyanobacteriales* and *Synechococcales* for the first three phases, but negatively correlated in the last phase (Fig. 7A), however, it was mostly negatively correlated with these cyanobacterial taxa in SH (Fig. 7B). These discrepancies indicated that the bacteria-cyanobacteria interaction may additionally be affected by environmental parameters.

### Correlations of cyanobacterial and bacterial communities with the environmental parameters

**Figure 8:**
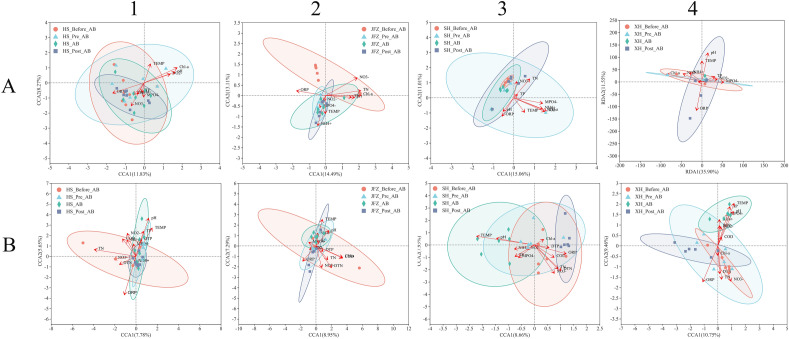
CCA/RDA analyses on cyanobacterial (**A1**–**A4**) and bacterial communities (**B1**–**B4**) of HS, JFZ, SH, and XH rivers at different cyanobacterial bloom phases.

The VIF analysis (Table S4) concluded that all parameters expect DTN, DTP, and COD were selected for cyanobacterial CCA/RDA, whilst all the parameters were used for bacterial CCA/RDA, and analyses were separately performed on microbial communities of each river at different phases (Fig. 8).

In general, cyanobacterial communities in these rivers at different phases were mainly correlated with ORP, pH, TEMP, MPO_4_^−^, NO_3_^−^ and Chl-*a*, and communities of each river had a specific correlation with additional parameters (Fig. 8A). For example, cyanobacteria in JFZ also had a close correlation with TP, TN, and NH_4_^+^ (Fig. 8A2), and the ones in SH had a close correlation with TN, NH_4_^+^, and NO_2_^−^ (Fig. 8A3). Additionally, the cyanobacterial communities of these rivers also exhibited variance in correlations with the main parameters, which indicated the discrepancy of the cyanobacterial communities amongst these rivers. For instance, most of the samples of HS across cyanobacterial bloom were positively correlated with ORP, TN, NH_4_^+^, NO_3_^−^, MPO_4_^−^, TP, TN, and were either not or negatively correlated with the rest main parameters, including pH, TEMP, NO_2_^−^, and Chl-*a*, whilst most samples in JFZ but the ones of “Before AB” showed a positive correlation with TEMP, ORP, NH_4_^+^, and MPO_4_^−^, and negative correlation with TN, NO_3_^−^, TP, pH, and Chl-*a*. Cyanobacterial communities in SH were universally negatively correlated with the main parameters, whilst the ones in XH largely illustrated a positive correlation with TEMP, pH, NO_3_^−^, and MPO_4_^−^. The correlation between XH communities and the main parameters was reflected through RDA according to the DCA analysis.

For bacterial communities in all rivers at different phases, they were mainly correlated with COD, TN, DTN, NO_3_^−^, ORP, TEMP, and pH, and communities of each river also showed specific correlation with additional parameters (Fig. 8B). Bacterial communities in HS additionally exhibited a close correlation with TP, NO_2_^−^ and Chl-*a* (Fig. 8B1), the counterparts in JFZ were with NH_4_^+^ and Chl-*a* (Fig. 8B2), the ones in SH and XH were both with TP, MPO_4_^−^ and NH_4_^+^ (Fig. 8B3), whilst XH showed additional correlation with DTP, and NO_2_^−^ (Fig. 8B4). Interestingly, different from cyanobacteria, the bacterial communities of these rivers demonstrated relatively consistent correlation patterns with the main parameters. For example, communities of “During AB” in these rivers universally showed a positive correlation with pH, TEMP, and TP, and negatively with TN, DTN, NO_3_^−^, and ORP, except HS, the rest three rivers also collectively showed negative correlations with Chl-*a*. Furthermore, communities of “During AB” in these rivers consistently exhibited contrasting correlation patterns compared with their counterparts either in “Post AB” or “Before AB”.

## Discussion

### Cyanobacterial bloom is a major factor influencing diversities of microbial community

The significance tests amongst the environmental parameters demonstrated that most of the parameters were not significantly different across the bloom phases (Table S1, *P* > 0.05), however, both the cyanobacterial and bacterial communities varied not only across the bloom phases in each individual river, but also amongst rivers at each of the same phases (Fig. S2). Additionally, more universal significant differences of cyanobacterial and bacterial communities were determined in rivers of JFZ, SH, and XH across the bloom (Fig. 2B,D), and the community diversity was significantly lower at the “During AB” phase (*P* < 0.05). The results as a whole indicate that the cyanobacterial bloom is a major factor affecting the diversities of both cyanobacterial and bacterial community.

It has been universally revealed that the main harmful algal blooms could significantly reduce the diversities and functions of other microbial communities, including coast and freshwater planktonic communities^5,6^, microbial communities associated with algae^18,19^, as well as those grew with macrophytes^20^, and sedimentary dwellers^7^. At the “During AB” phase, diversities of cyanobacterial community in JFZ, SH, and XH also significantly reduced, which inferred that the bloom was due to the overproduction of a narrower range of cyanobacterial taxa. Most studies concerning algal blooms focused on *Microcystis* and related species, which were ubiquitous in life-depending rivers and lakes in China, including Yangtze River, Taihu Lake, and Chaohu Lake^21–24^. It is therefore of importance to identify the dominant cyanobacterial taxa, their dynamic changes, and how the bacterial community were correspondingly affected along the course of cyanobacterial bloom in rivers of this study.

### The distinct spatial and temporal variance of cyanobacterial community composition along the bloom course

Instead of *Microcystis*, the dominant cyanobacterial taxa were *Cyanobacteriales* and *Synechococcales* both in the rivers and their tributary pools (Fig. 3A). Even though these water bodies are close in the locality and have similar environmental conditions, they revealed distinct cyanobacterial community compositions and patterns of dynamic changes in abundances along the bloom phases. Additional beta-diversity analyses on OTU level compared both different rivers at the same phase and each river along the bloom course, the results further illustrated that cyanobacterial community significances were shown in rivers at different bloom phases (Fig. 4A,B), especially for SH, and XH. Beside the bloom influence, the differences of community composition amongst rivers at each phase were also shown, indicating the spatial heterogeneity, which might be due to human activities and riverine characteristics as suggested in other studies^25,26^. Because of the relatively slow flowrate and large surface area, the tributary pools may therefore possess distinct cyanobacterial communities relative to the mainstream rivers, rendering the pools relatively independent ecosystems from rivers to some extent.

The dominant cyanobacterial species (OTUs) were further screened out through significance tests and LEfSe analyses on each river at different bloom phases (Fig. 5). The identified dominant species belonged to cyanobacterial genus of *Mychonastes*, *Trachydiscus*, *Oscillatoriales*, *Chamaesiphon*, *Nannochloropsis*, *Monoraphidium*, and *Cyanobium*, whose ecological functions included feeds for livestock^27,28^, constitutive members of biofilms^29,30^, biodiesel derivation^31,32^, and cyanobacterial early-stage bloom^12,33^. No dominant species were identified from rivers at the “During AB” phase, which may imply that relevant cyanobacterial OTUs in each river were relatively similar in abundance and composition during the bloom.

These results together may shed light on the ongoing treatment campaigns on eutrophication and algal blooms in China, that corresponding strategies should be taken into consideration when dealing with rivers and pools (ponds, reservoirs, and lakes). In general, the cyanobacterial bloom is the major factor that leads to the dynamic variation of community composition, which not only reduces the microbial diversities, assimilating cyanobacterial communities but also would affect the functions of relevant bacterial communities.

### Dynamic changes of dominant bacterial community, implications of their ecological functions and cyanobacteria-bacteria interaction

Collective comparisons of bacterial genera composition in each river at different phases and amongst rivers at the same phases revealed the spatial and temporal heterogeneity of bacterial communities (Fig. 3B–E). The dominant bacterial genus including *Acinetobacter*, CL500-29, *hgc*I clade, *Limnohabitans*, *Flavobacterium*, *Rhodoluna*, *Porphyrobacter*, *Rhodobacter*, *Pseudomonas*, and *Rhizobiales* could also be traced in other relevant studies. For example, in a study concerning Tianmuhu Lake, CL500-29 and *hgc*I clade were dominant genera and closely associated with cyanobacteria, whilst in the lake’s rivers, *Flavobacterium*, *Limnohabitans*, and *Rhodoluna* were the dominant genera^34^. *Porphyrobacter*, *Rhodobacter*, *Pseudomonas*, and *Rhizobiales* were also dominant concerning either cyanobacterial or *Microcystis* bloom in different aquatic ecosystems^35–39^.

The LEfSe analysis identified dominant bacterial species (OTUs) in each river at each phase, which demonstrated the dynamic responses of exact bacterial species to the bloom, implying their close interactions with the bloom-forming cyanobacteria (Fig. 6). For example, abundance of *Porphyrobacter* (OTU6265), *Rhodobacter* (OTU853 and OTU5851), and *Acinetobacter* (OTU2460, OTU2184, and OTU2685) were significantly higher at “During AB” phase. It was reported that species of *Acinetobacter* exhibited algicidal activity against *Microcystic aeruginosa*^40^, therefore, the outbreak of cyanobacterial bloom may also be likely to give rise to its adverse communities to keep the dynamic balance.

Furthermore, it is believed that the bloom-forming cyanobacteria have profound interaction with the bacterial communities, which has long been the research of interest^9–11,41,42^. Cyanobacterial species can act as bactericidal or growth-inhibiting factors, influencing the bacterial communities^18,35,43^, similar as various microbial agents act in other natural environments and artificial constructions^7,44^. On the other hand, various bacterial species, including those identified in this study, have also been reported to be cyanobacterial or growth-inhibiting factors^40,45–47^, which can be further studied to develop microbial reagents to alleviate or control the effects of cyanobacterial bloom. Additionally, many cyanobacteria species are integral members of biofilms in various environments^29,30,48^. These facts jointly reveal that cyanobacteria as a whole are indispensable part of the microbial community. Based upon this cognition, to alleviate or control the cyanobacterial bloom is to restore microbial equilibrium in ecosystems rather than to eliminate cyanobacteria, and understanding about the dynamic changes of cyanobacterial and bacterial communities, as well as those microbial taxa and dominant species relevant to each cyanobacterial bloom phase is taking one key step towards the comprehension of cyanobacteria-bacteria interactions and the behind mechanisms.

### Discrepancies of correlations between microbial communities and environmental parameters

The correlation patterns of cyanobacterial communities with the environmental parameters additionally indicated the discrepancies of community composition amongst samples at different bloom phases in each river (Fig. 7). Temperature, nitrogen, and phosphorus were universally considered to be the crucial factors affecting cyanobacterial growth and bloom^2,3,49,50^, and this study showed that environmental parameters, including temperature, nitrogen (ammonium, nitrate, etc.), and phosphorus (MPO_4_^−^ and TP), were also important to the cyanobacterial community compositions^2–4,51^, and parameters even with not significant difference (Table S1) could shape distinct cyanobacterial community.

The cyanobacterial bloom influenced the correlation patterns of bacterial communities of each river with the environmental parameters (Fig. 7B), that bacterial communities at the “During AB” phase generally had contrasting correlation patterns compared with the rest phases. The difference may infer that functional bacterial community on certain environmental parameters were additionally impaired due to the cyanobacterial bloom, and which metabolic pathways were impaired will be included in future studies.

## Conclusions

This study investigated the dynamic changes of cyanobacterial and bacterial communities in four upstream rivers, namely Huangshan River (HS), Jinfuzhai River (JFZ), Sihe river (SH), and Xihua River (XH), of a eutrophicated water source reservoir in the subtropical area of China. The major conclusions were drawn as follows.

- The bloom has a major influence on cyanobacterial and bacterial communities, rendering the alpha-diversities of bacterial communities significantly lower at “during cyanobacterial bloom” phase compared with other relevant phases;
- The studied rivers commonly shared dominant cyanobacterial taxa, including *Cyanobacteriales* and *Synechococcales*, and commonly shared several dominant bacterial genera, including *Acinetobacter*, *Flavobacterium*, *Rhodobacteraceae*, *Comamonadaceae*, etc. However, the corresponding compositions varied along with the bloom, making cyanobacterial communities significant difference amongst phases in SH and XH, and bacterial communities significant difference amongst phases in JFZ, SH, and XH;
- The studied rivers had distinct cyanobacterial and bacterial dominant species (OTUs) along with the bloom, and the bacterial dominant species with significantly higher relative abundances at “during cyanobacterial bloom” phase than other phases, including *Rhodobacter*, *Rhodobacteraceae*, *Porphyrobacter*, *Acinetobacter*, and *Pelomonas*, may indicate their close correlations with cyanobacterial bloom in each river;
- Cyanobacterial communities at different bloom phases in each river were mainly correlated with ORP, pH, TEMP, MPO_4_^−^, NO_3_^−^, and Chl-*a*, and the bacterial counterparts were mainly with ORP, pH, TEMP, COD, TN, DTN, and NO_3_^−^. However, communities of different rivers at different phases showed specific correlations with the above parameters.

The dynamic shifts of cyanobacterial and bacterial communities may imply the interactions between cyanobacteria and bacteria, to fully understand and verify the interaction, it is suggested that future studies should expand to network analysis and field experiments investigating the effects of bacterial species identified in this study on the cyanobacterial blooms.

## Supplementary Information

Supplementary Information 1.

Supplementary Information 2.

Supplementary Information 3.

Supplementary Information 4.

Supplementary Information 5.

Supplementary Information 6.

Supplementary Information 7.

## Data Availability

The original sequencing data was updated to the NCBI database under the accession number from SAMN23614736 to SAMN23614843. The direct link to the uploaded data is as follows: https://www.ncbi.nlm.nih.gov/sra?linkname=bioproject_sra_all&from_uid=786052.
