# Supplementary material for: Spatiotemporal changes of bacterial communities during a cyanobacterial bloom in a subtropical water source reservoir ecosystem in China
Source: Sci Rep. 2022 Aug 26;12:14573. doi: 10.1038/s41598-022-17788-7 (PMC9418230; doi:10.1038/s41598-022-17788-7)
Supplement: Supplementary file 1 — Supplementary Information 1. [file 41598_2022_17788_MOESM1_ESM.pdf]

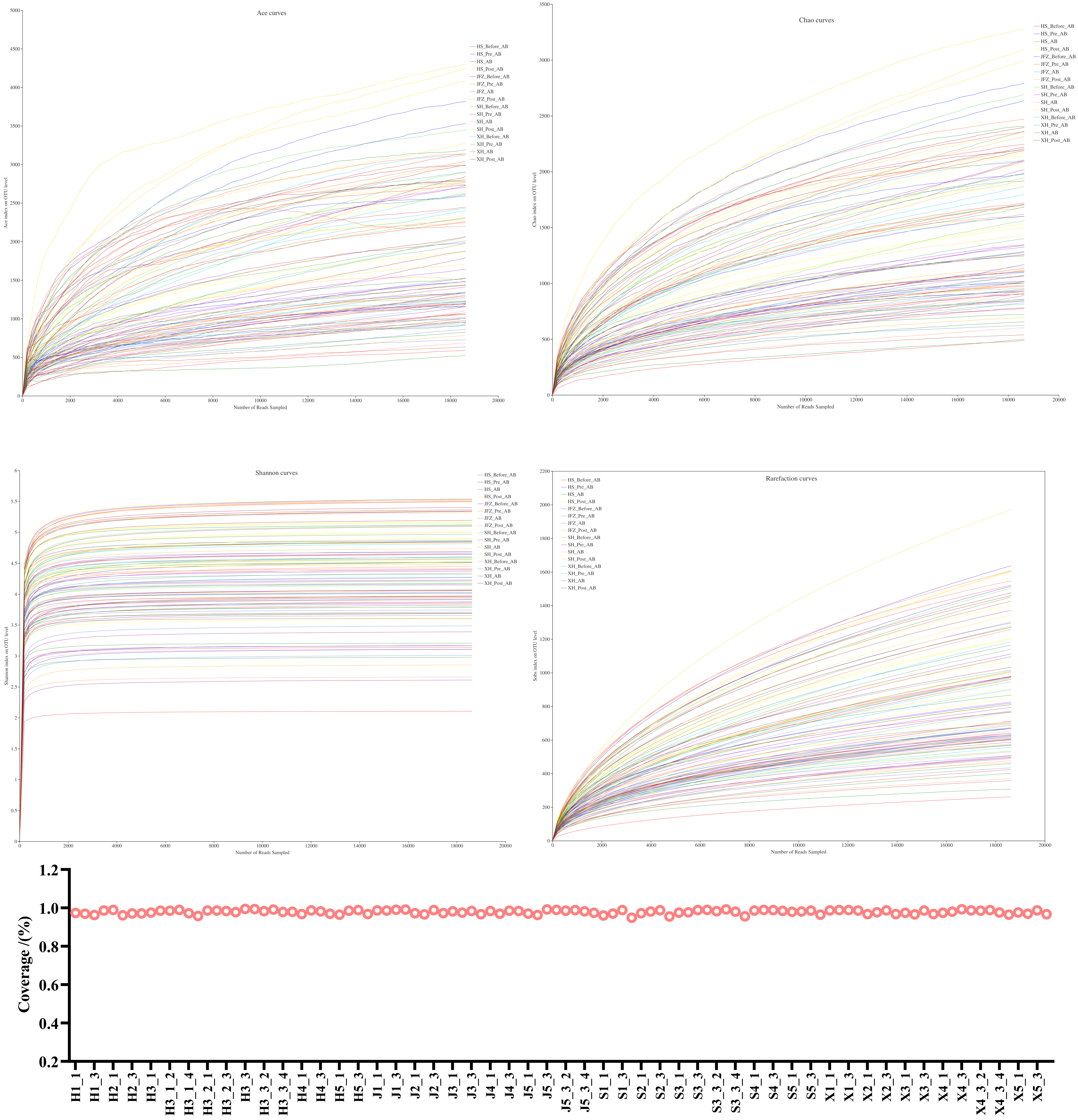

Figure S1. Rarefaction curves on alpha-diversity estimators of ACE, Chao1, Shannon and Sobs, and Good’s coverage of metagenomic sequencing on each sample.
