# Supplementary material for: Spatiotemporal changes of bacterial communities during a cyanobacterial bloom in a subtropical water source reservoir ecosystem in China
Source: Sci Rep. 2022 Aug 26;12:14573. doi: 10.1038/s41598-022-17788-7 (PMC9418230; doi:10.1038/s41598-022-17788-7)
Supplement: Supplementary file 2 — Supplementary Information 2. [file 41598_2022_17788_MOESM2_ESM.pdf]

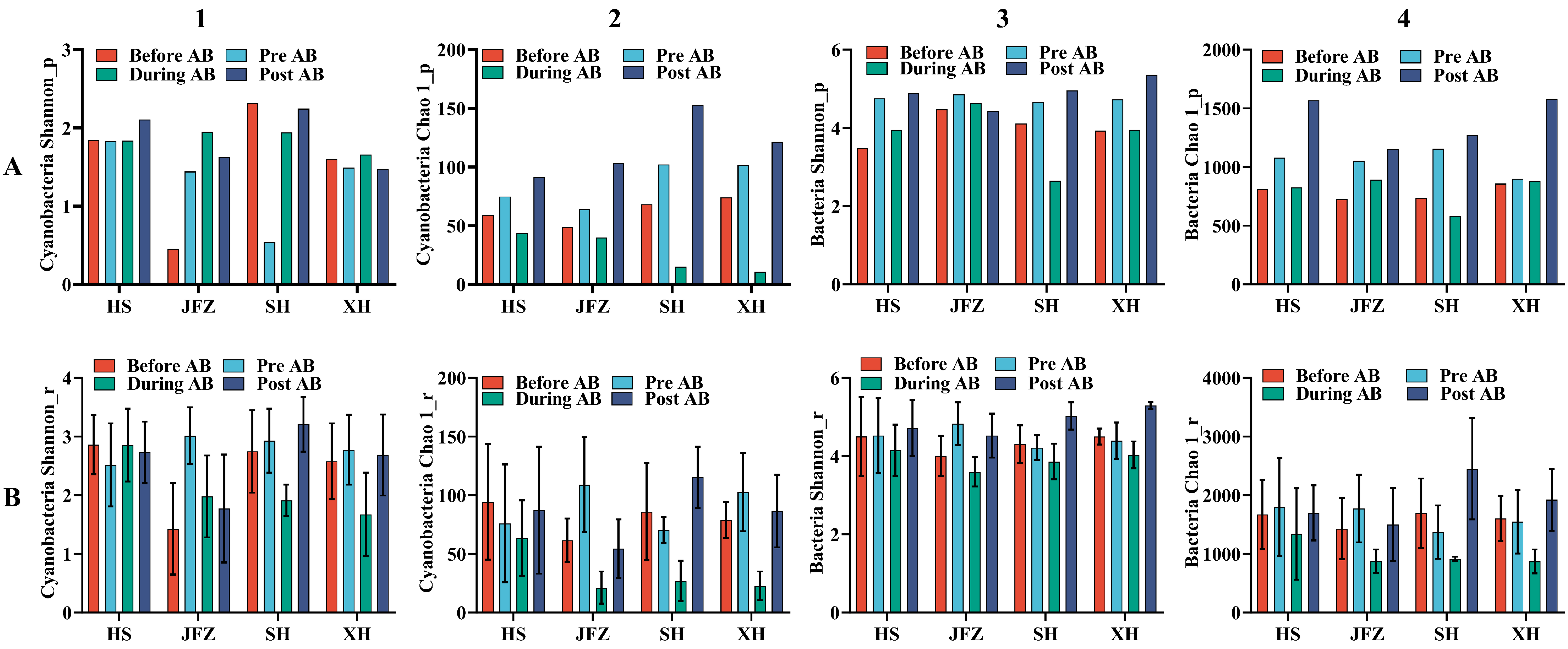

Figure S2. Alpha-diversity estimators of Shannon and Chao1 on cyanobacterial and bacterial communities in tributary pools (_p) and rivers (_r) were shown in mean values. Error bars represented standard deviation (SD) (n=8 for HS, and 6 for JFZ, SH, and XH, respectively).
