# Supplementary material for: Spatiotemporal changes of bacterial communities during a cyanobacterial bloom in a subtropical water source reservoir ecosystem in China
Source: Sci Rep. 2022 Aug 26;12:14573. doi: 10.1038/s41598-022-17788-7 (PMC9418230; doi:10.1038/s41598-022-17788-7)
Supplement: Supplementary file 3 — Supplementary Information 3. [file 41598_2022_17788_MOESM3_ESM.pdf]

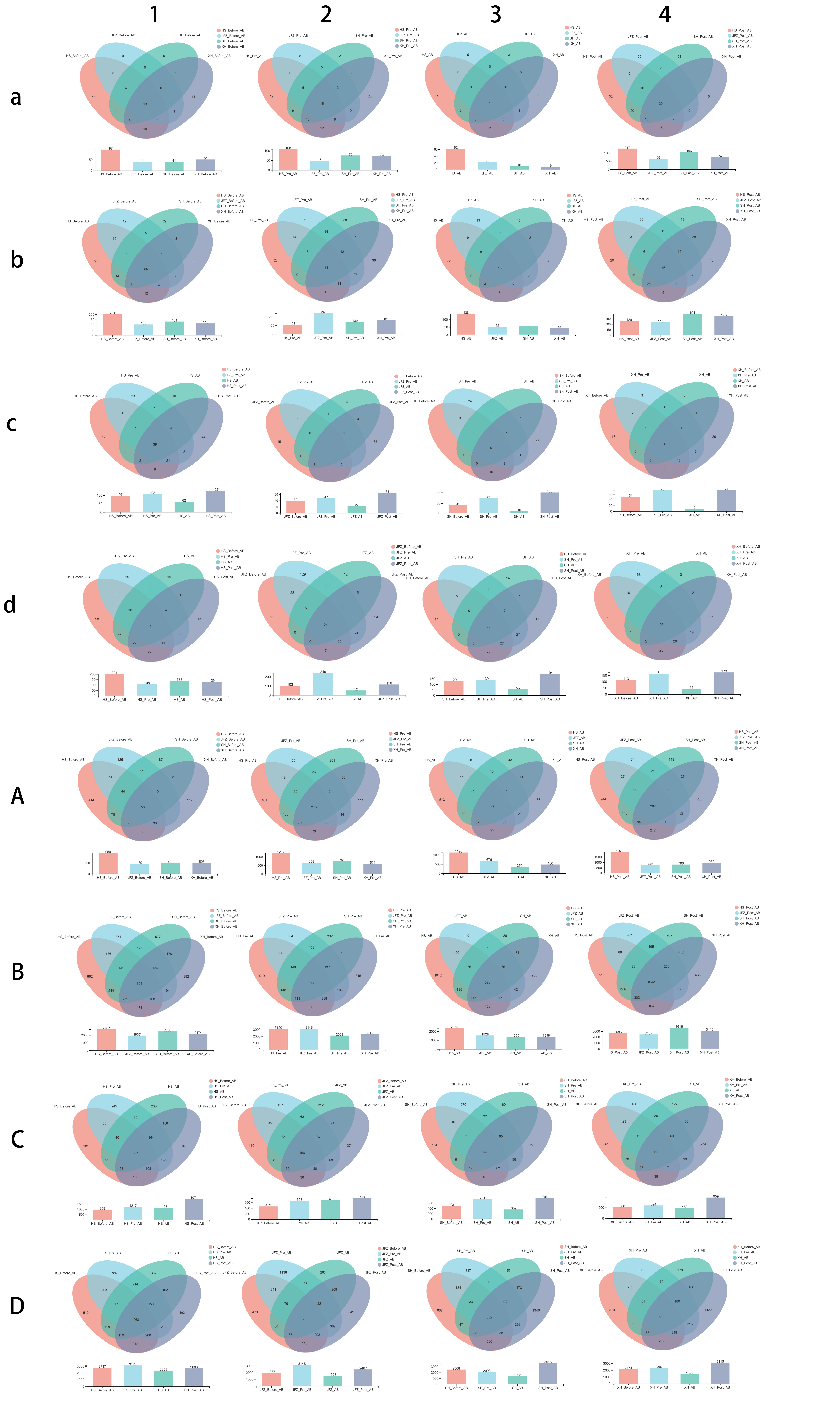

Figure S3. Venn diagrams comparing both cyanobacterial (a1-d4) and bacterial OTUs (A1-D4) of tributary pools and rivers at different bloom phases.
Figures of a1-a4 compared cyanobacterial OTUs of different tributary pools at the same phase of Before AB, Pre AB, During AB, and Post AB, and figures of b1-b4 showed cyanobacterial OTUs of HS, JFZ, SH, and XH at the same phase.
Figures of c1-c4 illustrated cyanobacterial OTUs of each tributary pool(s) at different phases, and figures d1-d4 showed the counterparts of each river at different phases.
Figures of A1-D4 stood for comparisons of bacterial community corresponding to a1-d4 of cyanobacteria.
