# Supplementary material for: Spatiotemporal changes of bacterial communities during a cyanobacterial bloom in a subtropical water source reservoir ecosystem in China
Source: Sci Rep. 2022 Aug 26;12:14573. doi: 10.1038/s41598-022-17788-7 (PMC9418230; doi:10.1038/s41598-022-17788-7)
Supplement: Supplementary file 4 — Supplementary Information 4. [file 41598_2022_17788_MOESM4_ESM.pdf]

#SampleID HS		TN	DTN	NH_4^+	NO_3^-	NO_2^-	TP	DTP	MPO_4^-	pH	TEMP	ORP	Chl-*a*	COD
Before AB	H1_1	3.03	2.60	0.00	2.16	0.01	0.12	0.03	0.04	7.85	20.30	-46.10	1.86	3.57
	H2_1	2.88	2.73	0.00	2.62	0.01	0.09	0.04	0.06	7.40	25.00	-20.70	2.79	3.65
	H3_1	2.85	2.46	0.64	2.40	0.02	0.15	0.05	0.08	7.87	22.40	-44.30	0.93	4.73
	H4_1	3.90	2.58	1.92	2.16	0.02	0.18	0.04	0.06	7.83	25.40	-49.00	0.00	3.85
	H5_1	3.82	2.35	1.88	2.16	0.02	0.18	0.07	0.06	7.78	25.20	-42.70	0.00	3.34
	H3_1_1	4.46	2.18	1.80	0.90	0.07	0.12	0.04	0.04	7.65	25.50	-35.10	3.72	3.46
	H3_2_1	2.38	2.18	1.34	1.21	0.05	0.27	0.02	0.02	9.04	24.50	-119.50	166.47	9.07
	H3_3_1	20.40	3.46	0.62	3.14	0.26	0.18	0.07	0.14	8.03	24.80	-63.60	107.88	7.53
Pre AB	H1_2	1.89	1.74	0.10	1.07	0.02	1.07	0.01	0.04	7.98	24.40	-53.30	37.94	1.95
	H2_2	2.68	2.47	0.89	1.33	0.05	1.33	0.03	0.06	7.99	25.20	-52.30	0.00	2.05
	H3_2	2.74	2.22	0.11	1.39	0.02	0.13	0.00	0.08	7.98	28.00	-53.60	1.12	2.39
	H4_2	3.18	2.91	0.12	1.48	0.12	0.18	0.10	0.10	8.30	31.90	-74.90	5.58	2.49
	H5_2	2.69	2.63	0.14	1.15	0.04	0.15	0.08	0.10	9.53	34.10	-148.80	17.86	3.12
	H3_1_2	1.14	0.99	0.10	0.38	0.02	0.09	0.05	0.04	8.55	31.20	-87.80	35.34	3.32
	H3_2_2	1.34	0.56	0.18	0.00	0.30	0.09	0.07	0.03	9.34	31.20	-137.80	325.50	7.71
	H3_3_2	2.79	1.76	0.15	0.67	0.47	0.22	0.20	0.16	9.10	31.10	-122.30	89.28	5.80
During AB	H1_3	3.46	3.16	0.22	0.92	0.07	0.43	0.05	0.09	7.92	28.50	-54.90	0.00	2.07
	H2_3	5.21	3.90	0.25	4.40	0.14	1.24	0.14	0.18	8.80	31.10	-104.70	0.00	2.73
	H3_3	3.01	2.06	0.34	2.15	0.16	4.81	0.17	0.20	9.33	33.10	-137.30	1.67	3.44
	H4_3	2.13	1.82	1.12	1.13	0.05	3.44	0.05	0.08	9.03	30.90	-117.90	1.12	3.62
	H5_3	2.07	1.56	0.25	1.44	0.07	1.58	0.07	0.11	9.29	31.70	-132.80	1.67	3.25
	H3_1_3	1.77	0.74	0.22	0.08	0.05	2.09	0.04	0.14	7.70	31.30	-38.90	1.86	3.20
	H3_2_3	1.80	0.92	0.31	0.07	0.05	0.43	0.05	0.00	9.36	32.10	-137.90	45.57	8.89
	H3_3_3	2.27	1.89	0.71	0.23	0.27	1.72	0.30	0.50	9.33	33.10	-137.30	19.53	8.56
Post AB	H1_4	2.75	2.54	0.13	1.30	0.00	0.09	0.05	0.06	8.14	22.40	-66.00	1.67	3.98
	H2_4	3.29	3.14	0.14	1.53	0.04	0.14	0.08	0.09	8.19	22.30	-67.60	1.12	5.87
	H3_4	3.09	2.68	0.25	1.32	0.07	0.22	0.16	0.16	7.71	22.80	-39.60	6.70	5.42
	H4_4	2.75	0.24	0.12	1.13	0.03	0.13	0.09	0.10	7.65	21.80	-36.30	5.58	4.62
	H5_4	3.96	2.68	0.12	1.16	0.03	0.14	0.10	0.10	7.75	22.10	-41.70	6.14	4.58
	H3_1_4	2.83	2.08	0.20	1.03	0.07	0.22	0.06	0.06	7.84	24.30	-47.60	1.67	3.49
	H3_2_4	2.57	2.00	0.18	0.71	0.02	0.28	0.06	0.07	9.11	23.70	-121.80	35.71	3.74
	H3_3_4	2.83	2.61	0.17	0.94	0.12	0.35	0.21	0.20	8.01	24.30	-57.70	62.50	2.67
Note		Not Significant (N.S.)	N.S.	N.S.	N.S.	N.S.	N.S.	N.S.	N.S.	N.S.	N.S.	Before vs Pre<br>Before vs During<br>Pre vs Post<br>During vs Post<br>($P<0.05$)	Before vs Pre<br>Before vs During<br>Pre vs During<br>Pre vs Post<br>($P<0.05$)	N.S.

#SampleID JFZ		TN	DTN	NH_4^+	NO_3^-	NO_2^-	TP	DTP	MPO_4^-	pH	TEMP	ORP	Chl-*a*	COD
Before AB	J1_1	1.00	0.78	0.00	0.57	0.01	0.04	0.01	0.00	7.91	23.60	-45.60	1.40	4.34
	J2_1	1.90	0.76	0.00	0.65	0.00	0.05	0.01	0.00	7.62	23.50	-33.70	0.00	3.35
	J3_1	1.80	1.00	0.00	0.74	0.01	0.09	0.01	0.02	7.58	21.60	-33.30	1.86	4.61
	J4_1	2.80	1.42	0.38	0.88	0.06	0.12	0.06	0.09	7.88	23.60	-50.30	0.00	3.43
	J5_1	10.68	1.22	0.00	0.24	0.04	0.10	0.04	0.07	7.70	28.40	-37.80	0.00	3.84
	J5_3_1	11.05	5.12	0.02	1.63	0.01	0.58	0.14	0.00	9.17	24.90	-128.60	535.68	31.74
Pre AB	J1_2	0.43	0.17	0.25	0.00	0.00	0.04	0.00	0.00	8.01	30.70	-60.40	3.72	3.22
	J2_2	0.67	0.57	0.13	0.19	0.01	0.09	0.01	0.03	7.63	26.00	-37.20	1.12	2.29
	J3_2	0.96	0.65	0.11	0.24	0.08	0.11	0.03	0.06	7.51	27.10	-30.90	2.23	2.49
	J4_2	2.27	2.22	0.11	1.02	0.01	0.15	0.11	0.13	7.82	27.20	-47.80	3.35	2.68
	J5_2	2.67	1.42	0.14	0.69	0.14	0.13	0.06	0.09	7.51	27.20	-27.10	16.74	3.07
	J5_3_2	1.48	1.45	0.15	0.62	0.01	0.11	0.08	0.06	9.04	30.30	-122.60	165.54	5.02
During AB	J1_3	0.64	0.32	0.31	0.02	0.00	0.02	0.00	0.00	8.72	32.50	-99.90	0.00	3.25
	J2_3	1.78	0.73	0.31	0.34	0.03	0.29	0.00	0.06	8.62	28.60	-93.10	0.00	2.78
	J3_3	1.15	1.13	0.37	0.68	0.09	0.49	0.04	0.13	8.67	30.30	-97.30	1.12	1.98
	J4_3	1.47	1.17	0.44	0.79	0.14	2.64	0.23	0.27	9.37	33.60	-139.90	0.56	2.73
	J5_3	0.57	0.39	0.34	0.00	0.02	0.28	0.07	0.01	7.57	31.70	-31.20	0.56	1.51
	J5_3_3	1.27	0.88	1.02	0.04	0.02	0.29	0.02	0.00	8.51	32.10	-88.70	9.49	7.86
Post AB	J1_4	0.47	0.37	0.19	0.01	0.01	0.04	0.00	0.01	7.76	24.30	-42.80	3.91	5.15
	J2_4	1.45	1.20	0.14	0.58	0.05	0.05	0.03	0.03	7.89	21.30	-50.00	0.56	3.71
	J3_4	1.80	0.07	0.19	0.84	0.16	1.88	0.10	0.08	7.92	22.20	-52.00	0.56	3.72
	J4_4	2.42	2.33	0.23	1.06	0.00	0.15	0.11	0.12	8.88	22.70	-108.20	59.15	4.12
	J5_4	2.22	2.12	0.50	0.95	0.06	0.08	0.04	0.05	7.13	22.20	-6.30	2.79	3.92
	J5_3_4	1.75	1.64	0.34	0.54	0.02	0.15	0.04	0.05	8.76	23.50	-101.30	18.97	7.83
Note		Not Significant (N.S.)	N.S.	N.S.	N.S.	N.S.	N.S.	N.S.	N.S.	N.S.	N.S.	N.S.	Before vs Pre<br>Before vs During<br>Before vs Post<br>($P<0.05$)	N.S.

#SampleID SH		TN	DTN	NH_4^+	NO_3^-	NO_2^-	TP	DTP	MPO_4^-	pH	TEMP	ORP	Chl-*a*	COD
Before AB	S1_1	3.64	2.58	1.26	1.38	0.01	0.06	0.03	0.02	7.50	22.90	-25.90	3.35	6.83
	S2_1	2.44	2.16	0.00	1.90	0.02	0.09	0.03	0.04	7.71	25.50	-38.50	0.00	3.46
	S3_1	2.85	2.48	0.00	2.73	0.02	0.14	0.03	0.05	7.74	24.70	-40.30	0.93	3.50
	S4_1	0.95	0.28	0.02	0.02	0.01	0.02	0.01	0.00	9.35	27.00	-135.70	2.09	4.77
	S5_1	2.15	1.96	0.00	1.75	0.04	0.26	0.12	0.21	9.10	24.40	-59.50	3.48	5.20
	S3_3_1	1.56	0.73	0.58	0.85	0.04	0.21	0.06	0.11	9.31	25.40	-132.20	3.90	4.39
Pre AB	S1_2	0.99	0.60	0.13	0.34	0.02	0.07	0.04	0.05	8.26	29.70	-72.10	0.00	2.39
	S2_2	1.46	0.92	0.15	0.29	0.01	0.06	0.03	0.04	8.44	31.40	-83.30	21.20	2.88
	S3_2	1.97	1.68	0.19	0.81	0.07	0.17	0.10	0.13	8.89	29.60	-108.80	2.23	3.85
	S4_2	0.21	0.03	0.16	0.00	0.01	0.02	0.00	0.01	8.69	32.10	-102.10	0.00	2.73
	S5_2	2.12	1.15	0.18	0.48	0.13	0.30	0.19	0.25	7.78	29.60	-46.90	7.81	3.46
	S3_3_2	1.21	0.87	0.83	0.18	0.52	0.31	0.28	0.27	8.13	30.40	-64.60	89.28	4.15
During AB	S1_3	1.83	1.24	0.30	1.14	0.09	1.10	0.07	0.41	8.78	33.60	-103.40	0.00	2.78
	S2_3	0.88	0.62	0.31	0.10	0.08	0.26	0.07	0.12	9.08	34.60	-123.10	0.00	2.12
	S3_3	0.97	0.89	0.35	0.38	0.08	0.43	0.07	0.10	9.23	30.80	-131.80	0.00	1.65
	S4_3	0.37	0.30	0.29	0.06	0.00	0.03	0.00	0.00	9.65	33.00	-94.60	8.37	2.26
	S5_3	1.81	1.71	0.36	1.11	0.29	6.15	0.02	0.38	9.06	31.90	-119.80	0.00	3.76
	S3_3_3	1.57	0.77	0.65	0.08	0.04	0.30	0.09	0.04	8.92	31.70	-111.50	13.02	3.95
Post AB	S1_4	1.87	1.36	0.11	0.78	0.00	0.16	0.04	0.05	7.77	22.50	-43.20	1.12	3.47
	S2_4	2.31	1.95	0.09	0.98	0.03	0.11	0.06	0.05	8.01	23.50	-57.40	1.12	3.95
	S3_4	2.86	2.55	0.15	1.25	0.02	0.15	0.11	0.11	8.24	24.50	-71.00	0.00	2.90
	S4_4	0.25	0.20	0.10	0.03	0.01	0.02	0.00	0.00	7.89	24.50	-10.60	2.23	2.51
	S5_4	3.96	2.81	0.13	1.33	0.00	0.28	0.23	0.23	7.80	22.90	-45.30	6.70	1.93
	S3_3_4	1.60	1.22	0.16	0.38	0.01	0.19	0.04	0.05	5.87	24.50	-166.50	10.04	4.70
Note		Not Significant (N.S.)	N.S.	N.S.	N.S.	N.S.	N.S.	N.S.	N.S.	N.S.	N.S.	Before vs During<br>Pre vs During<br>During vs Post<br>($P<0.05$)	Before vs Pre<br>($P<0.05$)	N.S.

#SampleID XH		TN	DTN	NH_4^+	NO_3^-	NO_2^-	TP	DTP	MPO_4^-	pH	TEMP	ORP	Chl-*a*	COD
Before AB	X1_1	3.42	1.48	1.08	0.95	0.01	0.07	0.01	0.00	9.01	27.00	-115.20	12.09	2.27
	X2_1	2.58	2.00	0.06	2.13	0.01	0.06	0.03	0.04	7.77	26.80	-42.70	0.00	2.59
	X3_1	2.85	2.52	0.00	2.32	0.01	0.08	0.04	0.05	8.12	25.60	-62.50	0.70	3.27
	X4_1	2.68	2.32	0.00	2.18	0.02	0.09	0.04	0.00	8.72	23.10	-96.30	0.00	3.33
	X5_1	2.53	2.02	1.00	1.93	0.02	0.14	0.05	0.10	9.00	27.50	-115.20	1.40	4.85
	X4_3_1	2.03	1.42	0.00	1.38	0.03	0.08	0.01	0.02	8.85	24.70	-104.90	0.70	4.62
Pre AB	X1_2	0.89	0.67	0.22	0.25	0.02	0.05	0.00	0.01	8.64	31.10	-95.20	3.35	2.49
	X2_2	1.63	1.26	0.10	0.67	0.06	0.08	0.04	0.06	7.82	27.00	-45.40	2.23	2.39
	X3_2	1.98	1.74	0.11	0.99	0.10	0.12	0.06	0.07	8.28	30.20	-73.30	1.12	3.12
	X4_2	1.75	1.53	0.14	0.68	0.10	0.14	0.07	0.10	8.03	27.90	-57.20	46.87	4.10
	X5_2	2.12	0.05	0.18	0.85	0.08	0.11	0.05	0.08	7.58	27.70	-31.30	2.23	3.85
	X4_3_2	1.67	1.54	0.20	0.59	0.28	0.19	0.07	0.06	8.34	30.80	-78.30	41.29	3.86
During AB	X1_3	0.64	0.55	0.27	0.27	0.00	0.29	0.00	0.03	8.76	33.10	-102.80	4.46	2.87
	X2_3	1.00	0.00	0.63	0.07	0.22	6.04	0.22	0.32	8.76	33.10	-102.80	0.00	4.66
	X3_3	2.13	1.51	0.33	1.42	0.10	0.58	0.09	0.14	9.42	32.20	-141.50	0.56	3.06
	X4_3	0.95	0.76	0.52	0.11	0.05	1.72	0.05	0.10	8.86	32.70	-108.60	3.35	4.38
	X5_3	2.55	2.07	0.35	1.67	0.11	2.40	0.11	0.24	8.78	31.00	-103.50	0.56	4.05
	X4_3_3	1.45	1.19	0.98	0.04	0.12	1.80	0.13	0.17	8.88	32.50	-110.30	8.37	4.19
Post AB	X1_4	0.48	0.37	0.15	0.05	0.00	0.04	0.01	0.00	7.88	23.90	-49.80	1.67	2.73
	X2_4	2.55	2.31	0.37	0.97	0.20	0.22	0.18	0.18	8.68	21.80	-95.70	2.23	3.46
	X3_4	2.98	2.48	0.19	1.23	0.02	0.13	0.09	0.10	8.36	21.70	-76.50	1.12	3.18
	X4_4	2.58	2.24	0.14	1.03	0.04	0.20	0.11	0.11	7.85	22.30	-48.10	3.35	4.24
	X5_4	2.76	2.43	0.54	1.21	0.07	0.17	0.12	0.13	8.24	22.60	-71.40	5.02	4.33
	X4_3_4	1.79	1.69	0.26	0.62	0.05	0.16	0.08	0.07	7.40	24.00	-22.10	5.02	4.52
Note		Not Significant (N.S.)	N.S.	N.S.	N.S.	N.S.	N.S.	N.S.	N.S.	N.S.	N.S.	Before vs Pre<br>Before vs During<br>Before vs Post<br>Pre vs During<br>During vs Post<br>($P<0.05$)	Before vs Pre<br>Pre vs During<br>Pre vs Post<br>($P<0.05$)	N.S.
